# Identification of Nutritional Targets in Spanish Children Belonging to the LAyDI Cohort for the Development of Health Promotion Strategies in the First Two Years of Life

**DOI:** 10.3390/ijerph18030939

**Published:** 2021-01-22

**Authors:** María Gómez-Martín, Begoña Domínguez, Miguel Gueimonde, Sonia González

**Affiliations:** 1 Area of Physiology, Department of Functional Biology, Faculty of Medicine, University of Oviedo, Julián Claveria, 33006 Oviedo, Spain; gomezmarmaria@uniovi.es; 2 Group Diet, Microbiota and Health, Instituto de Investigaciones Sanitarias del Principado de Asturias (ISPA), Avd. Roma, 33011 Oviedo, Spain; mgueimonde@ipla.csic.es; 3 Group Comprehensive Approach to Childhood Overweight, Instituto de Investigaciones Sanitarias del Principado de Asturias (ISPA), Avd. Roma, 33011 Oviedo, Spain; begoa.dominguez@gmail.com; 4 Spanish Association of Primary Care Pediatrics (AEPap), Avda. de Burgos, 28036 Madrid, Spain; 5 Department of Microbiology and Biochemistry of Dairy Products, Instituto de Productos Lácteos de Asturias, Consejo Superior de Investigaciones Científicas (IPLA-CSIC), Paseo Río Linares, 33300 Villaviciosa, Spain

**Keywords:** children, dietary pattern, nutrient intake, nutritional targets, vitamin D

## Abstract

The first 1000 days of life seem to represent the temporal window of opportunity for modulating some of the risk factors associated with the later development of pathologies. Nonetheless, the dietary pattern and nutritional status of children receiving complementary feeding is still understudied. We aimed to assess the food intake in children from the LAyDI cohort (Spain) at 18 and 24 months of age and evaluate this in relation to nutrient requirements and bioactive compound consumption. This was a prospective and multicenter study analyzing information from administered questionnaires about general characteristics and food frequency consumption in 426 children of 18 months and 336 of 24 months. The observed intake of vegetables, fruits, dairy, and eggs was lower than the recommendations in both periods, contrary to the consumption of meat, fish, and pulses. The consumption of energy and macronutrients was similar for all ages studied, with protein intake being slightly higher than the recommended values. Regarding micronutrients, practically the whole sample fell below the vitamin D requirements. In addition, the estimated daily intakes of vitamin E and iron, at 24 months, were below the recommended values for this population group. The mean intake of phenols was around 650 mg/day. Flavanol intake as well as both types of fiber decreased from 18 to 24 months. In conclusion, although these results have to be confirmed in other populations, it seems pertinent to propose the design of nutritional strategies aimed at increasing the intake of vitamins D and E as well as iron in Spanish children up to 2 years.

## 1. Introduction

Adequate nutrition during early life is essential to ensure children’s growth, health, and neurological development [1]. Scientific evidence substantiating the impact of early nutrition on the later development of non-communicable diseases, such as obesity, hypertension, and diabetes, is continuously growing [2,3]. Some studies have evidenced the impact of early feeding practice, such as breastfeeding duration and time of introduction of complementary food, on the risk of overweight and obesity [4,5]. Thus, the first 1000 days of life, beginning at conception and ending at 24 months, seem to represent the temporal window of opportunity for modulating the risk factors associated with the later development of these pathologies [2,6,7,8]. From birth to 6 months of age, the World Health Organization (WHO) recommends the use of exclusive breastfeeding to ensure the energy and nutrient requirements of the newborn [9]. However, from this moment, the complexity of the diet increases as different foods are progressively introduced into the infant diet, until a diet similar in composition to that of adults is achieved.

In Spain, most commonly, the introduction of new foods begins at around 6 months of age, so that by the age of 18 months, all the food groups have already been included in the infant diet [10]. Some authors have proposed that by the age of two, the tastes and food preferences that will condition the later diet and food preferences of the individual have already been determined [11]. Nonetheless, the dietary pattern and nutritional status of children receiving complementary feeding is still understudied [12,13,14,15]. The few epidemiological studies analyzing global diet quality in this population group have reported a high intake of protein in children’s diet, in Spain and other European countries [16,17,18,19], which may act as obesogenic risk factor [17]. On the micronutrient level, iron and fiber were identified as two important nutritional targets for children up to 12 months of age [20]. Furthermore, a high intake of sodium, added sugars, and saturated fatty acids has also been reported in children [21,22]. Beyond the nutritional components, in the last few years, the intake of non-nutritive biologically active components, such as fibers and (poly)phenols, have attracted the interest of the scientific community. Although the direct health benefits of these compounds are not as extensively studied as in adults, a higher intake of dietary fiber has been associated with improved overall diet quality among children [23]. Moreover, higher intake (poly)phenols and flavonoids has been inversely associated with obesity [24]. On the basis of scientific evidence regarding the association between polyphenol consumption and the modulation of intestinal microbiota composition or in the regulation of peptides related to the regulation of solid food intake in the adult population, it is reasonable to expect that these may also be some of the mechanisms that explain the link with body weight in adolescents [24].

Therefore, the investigation of the dietary habits of children in this age range is of great importance for the establishment of early-life health promotion strategies that improve later health [25]. In this context, we aimed at describing the food intake, the nutritional status, and the consumption of bioactive compounds of children from the LAyDI cohort (Spain) at 18 and 24 months of age to explore differences among regions, gender, anthropometrical factors, and breast-feeding.

## 2. Subjects and Methods

This analysis is part of a prospective longitudinal study (LAyDI; Lactancia Materna y Desarrollo Infantil, “Breastfeeding and Child Development”) including 1200 Spanish toddlers born between April 2017 and March 2018, visiting Primary Care Pediatricians from the PAPenREd research network. The initial sample was calculated from the 2015 census data published by the National Institute of Statistics [26] and selected at random by recruiting the first newborn to attend the consultation each month. Parents were informed of the objectives of the study and gave their written consent before enrollment. Ethical approval was obtained from the Ethics and Scientific Research Committee of the Principality of Asturias (nº 213/16).

The subjects were recruited across different Spanish regions, as it is described in Supplementary Figure S1. The geographical aggregation was carried out at posteriori according to previous studies [27] in order to facilitate the interpretation of the results. The northern and central areas were the major contributors to the study sample (70% of the total).

Information concerning dietary assessment, medical status, and basal characteristics was obtained at 18 and 24 months in a sample of 426 children at 18 months of age (response rate of 42.14%) and 336 at 24 months (response rate of 33.23%).

### 2.1. Nutritional Assessment

Infant’s dietary information was collected by means of a weekly and semi-quantitative food propensity questionnaire (FFQ) adapted from the pilot study for the Assessment of Nutrient Intake and Food Consumption Among Kids in Europe (PANCAKE) [28] for the Spanish population, thus including typical foods and traditional regional recipes. Furthermore, food diaries were designed through an online tool that included detailed dietary information grouped into 11 food groups according to European Prospective Investigation into Cancer (EPIC) classification (Supplementary Table S1) [29] and one extra group for processed infant food. The questionnaires were completed by children proxies who received it as an email. Detailed instructions for completing the food diary were included at the beginning of each food category and clarified by phone when necessary. The validated picture book developed by the PANCAKE consortium was used for portion size estimation according to EU-Menu recommendations. Special attention was paid to cooking practices, number and amount of ingredients used in each recipe, as well as questions concerning menu preparation (e.g., type of oil used, type of milk used), and other relevant information for the study, such as the consumption of skin-on fruit. Although the consumption of breast milk was recorded, it was not possible to quantify the volume ingested per feed, which implies an underestimation in the nutritional assessment of breastfed children. Food intake was converted into servings according to the infant portion sizes proposed by the Spanish “General Direction of Public Health and Consumption” in order to compare the adherence to dietary recommendations [30]. Moreover, the consumption of foods was transformed into energy and macro- and micronutrients using the food composition tables developed by the Centro de Enseñanza Superior de Nutrición Humana y Dietética (CESNID) [31]. Additionally, detailed information regarding the type of protein, lipid, or carbohydrate consumed was completed from the food composition tables published by the United States Department of Agriculture (USDA) [32]. The content of the major classes and subclasses of (poly)phenol in the foods consumed in the sample was calculated using the Phenol-Explorer database [33]. This tool contains detailed information of the polyphenol content in more than 400 European foods, mainly determined by high-performance liquid chromatography (HPLC), gas chromatography (GC), and capillary electrophoresis (CE) [33]. The major classes of fiber (soluble and insoluble types) were completed from Marlett et al. [34], and nutritional composition data of infant formula, cereal products, and complementary foods (mixed puree or snacks and desserts) were adapted from Gómez-Martín et al. [35].

### 2.2. Anthropometric Measures

Child height and weight were recorded to the nearest 0.1 cm and 0.1 kg, respectively, by pediatric nurses at the age of 18 and 24 months. Body mass index (BMI) was calculated as weight in kilograms divided by the square of height in meters and adjusted by child age and gender. BMI z-score was calculated relative to WHO Child Growth Standards [36] using the WHO ANTHRO Software for Calculating Anthropometry, Version 2.0 [37].

### 2.3. Statistical Analyses

Statistical analysis was performed using IBM SPSS 24.0 (IBM SPSS, Inc., Chicago, IL, USA). Goodness of fit to the normal distribution was checked by means of the Kolmogorov–Smirnov test. Overall, categorical variables were summarized as percentages, and continuous variables using means and standard deviations. Chi-square test and independent samples Student *t*-test were used for intragroup comparisons and Bonferroni multiple comparison. Differences in general characteristics and food and nutrient intake were analyzed according to the geographical location of participants and age group. GraphPad Prism 8 and Biorender were used for graphical representations. Adherence to Dietary Reference Values (DRVs) was calculated using the European Food Safety Authority (EFSA) recommendations for children aged 1–3 years [38,39]. Specifically, the parameters used were average requirement (AR), adequate intake (AI), and reference intake range (RI) for 1–3-year-olds. AR was determined for calcium, iron, zinc, folate, niacin, riboflavin, thiamine, vitamin A, vitamin B_6_, and vitamin C, while AI was determined for phosphorus, magnesium, manganese, vitamin B_12_, vitamin E, and vitamin D. The AI can also be used to determine the proportion of individuals with adequate nutrient intake.

## 3. Results

### 3.1. Description of the Sample

The general characteristics of the study sample according to age are listed in Table 1. The gender-balanced samples consisted of a total of 426 and 336 subjects, at 18 and 24 months of age, respectively. Almost the whole sample had received the prescribed vaccinations from birth to 18 months of age, did not report health problems in the 6 months prior to the interview, and slept between 11 and 14 h/day. Regarding dietary habits, despite most of the volunteers following a normal diet at 18 and 24 months, significant variations in its texture were observed across ages. The percentage of children consuming a semi-solid diet decreased from 45.2% at 18 months to 28% at 24 months of age. In addition, our data reveal that over 30% of the children were still breastfeeding. Concerning anthropometric parameters, the percentage of children with normal weight (BMI z-score from −1 to 1) decreased significantly from 18 to 24 months (70.9 to 63.7%).

### 3.2. Dietary Intake and Related Factors

The intake of the major food groups was described in the sample and analyzed according to gender, region, breastfeeding, and BMI z-score ranges at 18 and 24 months (Table 2). The obtained results point to the absence of differences in food consumption for both ages by gender and region; hereafter, the analyses were conducted for the whole sample. The only differences observed by region were a higher intake of vegetables and lower intake of meat and fish in the south and the Balearic islands, respectively, at 18 months, and differences in the consumption of eggs and fruits at 24 months on the Balearic islands and in southern Spain. Concerning breastfeeding, children receiving breast milk reported a lower intake of sweets and a higher intake of eggs at 18 months and a lower intake of potatoes, meat, and fish at 24 months, compared to those who were not breastfed. As expected, dairy product consumption, excluding breast milk, was higher in non-breastfeeding groups independent of age (Table 2, (A,B)). During the period of study, the consumption of fruits, vegetables, potato and tubers, and processed infant products decreased, and the consumption of meat and meat products, eggs, cereals and cereal products, and sweets and desserts increased (Table 2, (A,B)).

**Figure 1:**
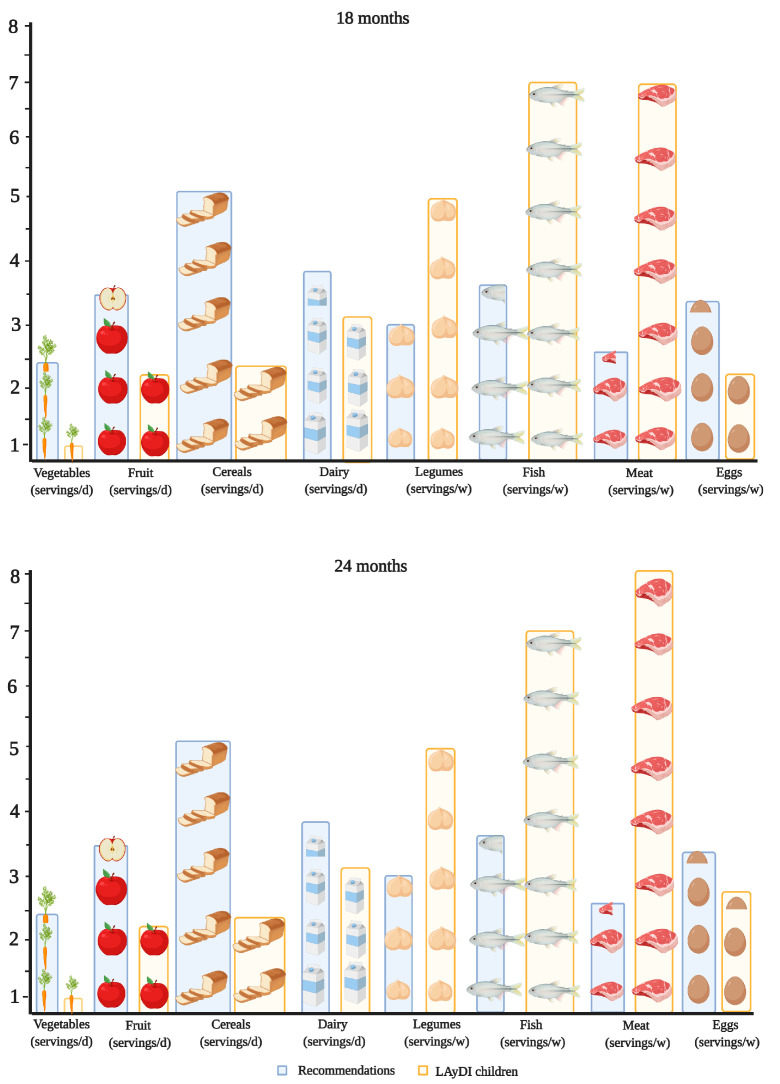
Adherence to dietary recommendations in the sample. D, day. W, week.

In order to evaluate the proportionality of the diet consumed, the average serving of the major food groups at 18 and 24 months was compared with the available recommendations for the infant population. The presence of vegetables, fruits, dairy, and eggs in the children’s diet was lower than recommendations, contrary to the consumption of meat, fish, and pulses in both periods (Figure 1).

### 3.3. Nutritional Targets in Children

**Figure 2:**
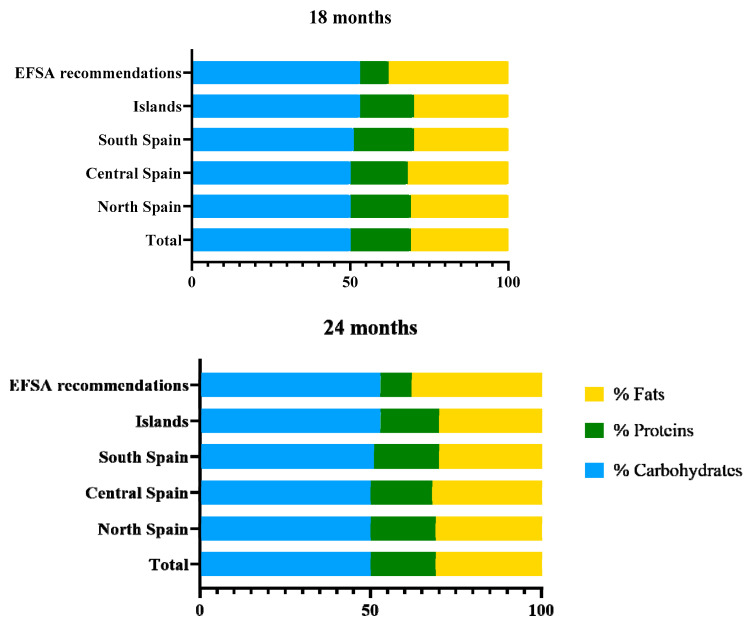
Percentage of the total energy intake provided by each macronutrient in the sample by region at 18 and 24 months. Mean daily energy intake at 18 months: total sample 1093.4 kcal; north Spain 1067.8 kcal; central Spain 1122.6 kcal; south Spain 1122.2 kcal; the islands 1053.9 kcal. Mean daily energy intake at 24 months: total sample 1094.2 kcal; north Spain 1093.30 kcal; central Spain 1117.6 kcal; south Spain 1083.2 kcal; the islands 1064.7 kcal.

A detailed description of the infant’s nutritional status, as well as a comparison with the recommended daily values by age is presented in Table 3. No significant differences by age were observed for energy or macronutrients. Nevertheless, it was revealed that at 24 months of age, fiber intake was lower than at 18 months, whereas the intake of monounsaturated fatty acids was higher. A moderate excess in protein intake was observed in both groups (Figure 2). Regarding micronutrients, the intake of magnesium, iron, and vitamin E were lower than the recommended values in the P_25_ of the sample at 18 and 24 months. It is remarkable that the whole sample is under the recommended intake of vitamin D at 18 and 24 months.

### 3.4. Bioactive Compounds

The average intake of the major classes and subclasses of (poly)phenols and fiber is presented in Table 4. Total phenols intake in the sample was 659.4 mg/day at 18 months and 654.2 mg/day at 24 months, with flavonoids and phenolic acids being the best contributors to their intake independent of age (data not shown). The intake of flavonols, total fiber, and insoluble and soluble fiber decreased with age.

## 4. Discussion

This study represents the first approach describing the dietary intake and nutritional status associated with complementary feeding in a representative sample of 18- and 24-month-old children from a Mediterranean country. Considering the health impact that nutritional habits at this life-stage may have, the knowledge gained from these results may be useful in the design of future strategies focused on the prevention of non-communicable diseases for the pediatric age group.

As expected, from the age of 18 months, all food groups were present in the toddlers’ diets. However, in contrast to what has been published on the adult population, no significant differences in food consumption were observed according to regions or gender at any age. In accordance with previous data in the literature, describing a nutrient-poor diet in children who are breastfed for a short time [40,41], a higher consumption of sweets in non-breastfed children at 18 months was observed. Surprisingly, a positive association was found between BMI z-score and vegetable consumption. Vegetables at this age are the basis of feeding and are also used as an accompaniment to the rest of the food groups. Thus, it is probable that this result reflects the consumption of a larger portion size in an indirect way.

It is noteworthy that the percentage of children that consumed vegetables (data not shown), fruits, and infant cereals was slightly higher than that observed in other studies of the same age group [42,43]. Interestingly, with the transition to 24 months, a reduction in the intake of fruits and vegetables (the major fiber sources in the sample) was observed in favor of meat and meat products, which is closer to the adult diet characteristic of westernized countries. On the basis of the estimated portion sizes for the 2-year-old pediatric population, the children in the sample had a clear excess of almost all high-protein foods, such as meat and meat products, fish and seafood, and legumes. In contrast, vegetables, fruits, and dairy products were less present in the diet than indicated. Although egg consumption was below the recommended frequency, it may have been underestimated, since egg is often used as an ingredient in the preparation of other products and, therefore, is difficult to record through an FFQ.

In consequence, the contribution of macronutrients to total energy intake was similar to previous studies, showing a moderate increase in protein (18% at both ages) and lipids (31% at 18 months and 32% at 24 months) as compared to the recommended values (Figure 2) [44,45,46,47]. This profile probably represents the characteristic energy distribution of adults in westernized countries [48]. Some of the differences observed with respect to other studies in children, such as ALSALMA [19] and DONALD [49], may be due to several factors including (i) the different methodology for dietary assessment; (ii) the wide age ranges compared, as few studies strictly adhere to 18 months; (iii) the variations in dietary habits among the studied populations. Our sample showed a lower intake of total and saturated fatty acids (37.3 and 14.4 g/day at 18 months and 38.3 and 15.5 g/day at 24 months) and a higher intake of monounsaturated fatty acids (16.5 g/day at 18 and 17.7 g/day at 24 months), compared to other studies carried out in non-Mediterranean countries [50,51,52], which is consistent with the use of olive oil as the main culinary fat in our population [53].

To elaborate, certain micronutrients, such as folate, vitamin D, iron, and calcium, have a key role during early life [8]. Regarding vitamins, folate intake in our study was higher than that shown in Norwegian toddlers at 24 months of age [54] and in the US Feeding Infants and Toddlers Study (FITS) between 12 and 24 months of age (555.3 and 496.9 µg/day vs. 87 and 308 µg/day, respectively) [55]. This increase in the average intake in our sample may be of interest because of the role of folic acid in the nervous system and blood-forming organs [56]. One of the most striking results in this work is the low intake of vitamin D in the sample. Despite calcium intake in the P_25_ being slightly above the recommended value (440.9 and 418.7 mg/day), vitamin D improves its intestinal absorption, promoting the mineralization of the bone matrix [57]. The determination of vitamin D intake is dependent on the information collected in food composition tables and is subject to wide seasonal variation. The intake of this vitamin is consistently low throughout the literature [58,59,60]; therefore, it seems reasonable to propose it as a target for intervention in this population group. For this purpose, in order to define vitamin D deficiency accurately, the serum determination of 25(OH) vitamin D and the recording of information relating to the child’s skin color, frequency of sun exposure, and supplementation would be advisable. Furthermore, since 30% of children are below the AI of Mg at 24 months, the levels of this mineral, which plays an essential role in bone metabolism, should be monitored together with calcium and vitamin D.

As regards minerals, iron intake is essential for the maintenance of the energy of the neural cells and the homeostasis of the neurotransmitters [8,61]. In accordance with various existing studies that reveal a low intake of this mineral [59,62], the average intake in the P_25_ of the sample was slightly lower than EFSA recommendations. This may be explained by the fact that the intake of processed baby foods, usually fortified with iron, decreased from 18–24 months.

To our knowledge, this is the first study describing the intake of bioactive compounds in the Spanish infant population. Although there are only a few publications with which these results can be compared, (poly)phenol intake was slightly higher than in other European cohorts [63] and similar to that reported in children from other Mediterranean populations, both in the average amount consumed [64] and in the contribution of the individual classes to the total intake [65,66]. In both age groups, the main dietary source of anthocyanins was strawberries, flavanols were provided by apples, bananas, pears, and lentils, and flavanones were derived mainly from oranges (data not shown). This is to be expected, since foods such as coffee, tea, and alcoholic beverages, which represent a significant portion of the polyphenol intake in the adult population, are not consumed in this age range. Different surveys in developed countries have identified fiber as one of the dietary components whose intake should be increased in the adult population. The results presented in this study address a gap in the literature as regards the intake of fiber in children under 2 years old, including the different types of fiber consumed, which can be related to different physiological effects. In this sense, the daily dietary fiber in our sample oscillates around 15 g/day, which is superior to what is reported in the majority of the literature [19,55,67] and can be adapted to the values recommended by the EFSA for this compound [68].

The study has some limitations inherent to its observational nature and the collection of dietary information. When interpreting this information, it should be considered that the energy and nutrient content of processed baby foods was considered, a factor that has been underestimated in the literature so far. Moreover, the Phenol-Explorer database used in the present study allowed us to provide information on (poly)phenol intake with a level of detail that had not been achieved before in Spanish children. However, at the time of analyzing the intake of bioactive compounds, it was not possible to incorporate the content of processed baby foods, since information concerning the type of fiber or (poly)phenol content was not present on the product label. Furthermore, we were not able to evaluate (poly)phenols from spices, even though these are a good dietary source of these compounds, since these data had not been collected in the FFQ. Although the quality of the FFQ depends on the memory of the respondent, its ability to accurately classify energy and all nutrient intakes in infants is enhanced by the quality and detail of the information collected [69]. The use of the methodology and questionnaires developed by PANCAKE allows for comparison with other studies on the European child population.

## 5. Conclusions

This work addresses certain gaps in the knowledge concerning infant nutrition and provides key information for the design of health promoting strategies for toddlers. Our results allowed us to affirm that the introduction of complementary feeding is similar throughout the Spanish national territory and is, in general, adequate in terms of the variables studied. It seems advisable to monitor protein consumption and to promote the maintenance of breastfeeding until 18 months of age, because of its association with a lower consumption of certain foods related to the risk of child obesity and related pathologies. Finally, studies specifically designed to analyze vitamin D deficiency in children and its impact on bone mineral balance would be of great interest.

## Figures and Tables

**Table 1:** General characteristics of the LAyDI cohort by age.

Characteristics		18 Months (N 426)	24 Months (N 336)
Gender	Boys	213 (50)	170 (50.6)
	Girls	213 (50)	166 (49.4)
Health status ^1^			
Complete immunization	Yes	422 (99.3)	-
Illness in the last 6 months	No	415 (97.6)	-
Sleep duration (hours/day)	<11	46 (10.8)	-
	11–14	337 (79.3)	-
	14–16	41 (9.6)	-
	>16	1 (0.2)	-
Diet			
Special diet ^2,3^	Yes	29 (6.9)	12 (3.6) *
Diet consistency ^1^	Mashed foods	3 (0.7)	1 (0.3)
	Semi-solid	192 (45.2)	94 (28) *
	Regular	230 (54.1)	241 (71.7) *
Breastfeeding ^1^	Yes	141 (33.2)	95 (28.4)
Anthropometric data			
Weight (kg) ^1,4^		10.9 ± 1.2	12.3 ± 1.6 *
Height (cm) ^1,3^		81.6 ± 3.2	86.9 ± 4.8 *
BMI z-score ^1,4^	Underweight (−5.0 to <−1)	54 (12.7)	52 (16.1)
	Normal weight (−1 to 1)	302 (70.9)	214 (63.7) *
	Overweight (>1 to 2)	57 (13.4)	54 (16.1)
	Obese (>2)	12 (2.8)	14 (4.2)

Data are presented as mean ± standard deviation or n (%). ^1^ N 425, ^2^ N 423 at 18 months and ^3^ N 335, ^4^ N 334 at 24 months. * *p* value for difference between 18 and 24 months from *t*-test for continuous variables and from chi-square test for categorical variables.

**Table 2:** Major food intake in the LAyDI cohort according to gender, region, breastfeeding (BF), and body mass index (BMI) at 18 (A) and 24 months (B).

(A)		Oils (mL/Day)	Vegetables (g/Day)	Legumes (g/Day)	Fruit (g/Day)	Potato and Tubers (g/Day)	Cereals and Cereal Products (g/Day)	Meat and Meat Products (g/Day)	Fish (g/Day)	Eggs (g/Day)	Processed Infant Products ^1^	Milk and Dairy Products ^2^	Sweets and Desserts (g/Day)
Total		12.2 ± 5.4	131.8 ± 112.9	23.8 ± 19.5	237.4 ± 149.8	26.3 ± 19.9	67.8 ± 40.3	33.8 ± 25.7	46.5 ± 31.5	20.2 ± 8.8	183.8 ± 229.0	340.4 ± 246.7	15.1 ± 15.7
Gender	Boys	12.2 ± 5.6 ^a^	135.3 ± 121.4 ^a^	24.4 ± 20.2 ^a^	238.9 ± 143.2 ^a^	28.1 ± 20.5 ^a^	66.7 ± 39.0 ^a^	34.0 ± 28.1 ^a^	46.8 ± 30.9 ^a^	19.7 ± 8.6 ^a^	176.2 ± 208.0 ^a^	354.9 ± 259.6 ^a^	15.2 ± 12.0 ^a^
	Girls	12.2 ± 5.2 ^a^	128.4 ± 103.8 ^a^	23.2 ± 18.9 ^a^	236.0 ± 156.4 ^a^	24.5 ± 19.0 ^a^	68.9 ± 41.5 ^a^	33.6 ± 23.2 ^a^	46.1 ± 32.1 ^a^	20.8 ± 9.0 ^a^	191.4 ± 248.8 ^a^	325.6 ± 232.5 ^a^	15.0 ± 17.5 ^a^
Region	North	11.8 ± 5.0 ^a^	127.4 ^a^ ±110.9 ^a,b^	23.0 ± 18.9 ^a^	238.3 ± 138.0 ^a^	27.2 ± 20.0 ^a^	65.5 ± 38.4 ^a^	37.2 ± 28.4 ^a^	47.3 ± 29.2 ^a^	20.4 ± 8.9 ^a^	183.6 ± 217.3 ^a^	321.4 ± 295.0 ^a^	15.3 ± 16.5 ^a^
	Central	12.6 ± 6.2 ^a^	116.8 ± 91.5 ^a^	21.3 ± 18.0 ^a^	244.7 ± 170.6 ^a^	25.0 ± 20.7 ^a^	67.8 ± 35.4 ^a^	33.5 ± 24.5 ^a,b^	44.5 ± 30.3 ^a,b^	20.8 ± 9.1 ^a^	172.4 ± 235.2 ^a^	385.0 ± 263.9 ^a^	16.3 ± 16.2 ^a^
	South	12.7 ± 5.8 ^a^	163.5 ± 144.3 ^b^	28.0 ± 21.8 ^a^	215.8 ± 140.5 ^a^	27.9 ± 20.9 ^a^	71.6 ± 44.2 ^a^	31.6 ± 24.5 ^a,b^	55.6 ± 36.8 ^a^	18.8 ± 7.1 ^a^	195.9 ± 231.4 ^a^	303.3 ± 201.9 ^a^	14.3 ± 10.5 ^a^
	Islands	11.0 ± 3.1 ^a^	135.1 ± 102.8 ^a,b^	26.3 ± 20.9 ^a^	253.9 ± 146.8 ^a^	22.9 ± 13.8 ^a^	70.4 ± 53.8 ^a^	23.5 ± 13.7 ^b^	30.6 ± 27.2 ^b^	20.5 ± 11 ^a^	201.7 ± 266.3 ^a^	354.0 ± 244.5 ^a^	12.5 ± 12.9 ^a^
BF	No	12.1 ± 5.3 ^a^	133.5 ± 111.8 ^a^	24.2 ± 20.1 ^a^	247.0 ± 152.7 ^a^	26.8 ± 19.3 ^a^	69.7 ± 40.6 ^a^	35.2 ± 25.5 ^a^	48.4 ± 32.0 ^a^	19.6 ± 8.5 ^a^	188.0 ± 237.3 ^a^	416.9 ± 243.4 ^a^	16.2 ± 16.4 ^a^
	Yes	12.3 ± 5.6 ^a^	128.8 ± 155.9 ^a^	22.9 ± 18.3 ^a^	219.2 ± 142.5 ^a^	24.9 ± 20.9 ^a^	63.5 ± 38.8 ^a^	31.0 ± 26.0 ^a^	42.9 ± 30.0 ^a^	21.5 ± 9.5 ^b^	170.9 ± 203.2 ^a^	184.0 ± 167.6 ^b^	12.4 ± 11.0 ^b^
BMI	−5 to −1	12.5 ± 6.2 ^a^	104.7 ± 96.3 ^a^	23.8 ± 17.8 ^a^	217.3 ± 141.0 ^a^	23.8 ± 17.1 ^a^	66.6 ± 40.0 ^a^	31.5 ± 21.9 ^a^	43.5 ± 30.3 ^a^	22.1 ± 8.8 ^a^	181.3 ± 249.5 ^a^	30.7 ± 238.6 ^a^	14.3 ± 10.5 ^a^
	−1 to 1	12.3 ± 5.5 ^a^	121.2 ± 102.3 ^a^	24.0 ± 19.3 ^a^	242.5 ± 154.4 ^a^	26.3 ± 19.9 ^a^	68.2 ± 39.9 ^a^	35.2 ± 27.6 ^a^	47.3 ± 31.9 ^a^	19.9 ± 8.9 ^a^	187.8 ± 225.7 ^a^	304.5 ± 251.0 ^a^	15.3 ± 16.6 ^a^
	1 to 2	11.5 ± 4.2 ^a^	155.0 ± 149.4 ^a,b^	23.5 ± 23.7 ^a^	219.4 ± 120.5 ^a^	26.6 ± 22.0 ^a^	62.9 ± 38.3 ^a^	28.5 ± 19.0 ^a^	46.7 ± 32.6 ^a^	21.5 ± 8.5 ^a^	163.3 ± 240.3 ^a^	383.6 ± 241.8 ^a^	15.4 ± 10.9 ^a^
	> 2	10.9 ± 3.0 ^a^	250.6 ± 159.5 ^b^	20.5 ± 12.9 ^a^	270.0 ± 189.5 ^a^	33.1 ± 21.5 ^a^	87.8 ± 57.7 ^a^	35.6 ± 17.2 ^a^	36.6 ± 22.0 ^a^	16.5 ± 8.4 ^a^	213.2 ± 209.4 ^a^	320.6 ± 192.3 ^a^	13.9 ± 10.5 ^a^
**(B)**													
Total		12.5 ± 5.3	107.8 ± 88.6	24.2 ± 19.0	177.5 ± 118.0	23.0 ± 16.8	75.4 ± 44.9	43.4 ± 25.5	45.4 ±29.4	22.7 ± 9.8	128.3 ± 192.7	356.1 ± 223.0	22.7 ± 24.5
Gender	Boys	12.4 ± 5.5 ^a^	109.7 ± 90.0 ^a^	26.0 ± 21.3 ^a^	196.0 ± 121.0 ^a^	24.2 ± 16.2 ^a^	70.9 ± 37.9 ^a^	43.4 ± 26.8 ^a^	43.6 ± 28.1 ^a^	23.4 ± 9.9 ^a^	117.0 ± 174.1 ^a^	355.9 ± 238.0 ^a^	25.14 ± 28.5 ^a^
	Girls	12.5 ± 5.1 ^a^	105.8 ± 87.4 ^a^	22.3 ± 16.0 ^a^	191.9 ± 94.0 ^a^	21.6 ± 17.3 ^a^	80.0 ± 50.7 ^a^	43.3 ± 24.2 ^a^	47.1 ±30.6 ^a^	22.0 ± 9.6 ^a^	140.4 ± 211.6 ^a^	356.4 ± 207.8 ^a^	20.2 ± 19.5 ^a^
Region	North	12.4 ± 5.0 ^a^	113.2 ± 94.4 ^a^	25.7 ± 21.7 ^a^	198.0 ± 125.1 ^a,b,c^	24.3 ± 16.3 ^a^	75.5 ± 44.2 ^a^	47.0 ± 28.1 ^a^	46.0 ± 28.1 ^a^	23.0 ± 10.8 ^a,b^	148.7 ± 211.0 ^a^	339.2 ± 228.4 ^a^	22.3 ± 27.2 ^a^
	Central	12.4 ± 5.3 ^a^	108.9 ± 90.8 ^a^	22.3 ± 17.0 ^a^	199.6 ± 95.8 ^a,b^	19.7 ± 13.2 ^a^	77.2 ± 41.9 ^a^	44.4 ± 25.4 ^a^	46.7 ± 29.5 ^a^	22.0 ± 8.6 ^a,b^	80.6 ± 134.6 ^a^	395.7 ± 228.6 ^a^	25.3 ± 24.8 ^a^
	South	11.8 ± 5.4 ^a^	82.6 ± 59.9 ^a^	24.8 ± 15.3 ^a^	159.9 ± 76.9 ^c^	25.01 ± 22.1 ^a^	86.3 ± 52.9 ^a^	41.1 ± 17.6 ^a^	46.6 ± 27.1 ^a^	20.1 ± 8.9 ^a^	109.8 ± 157.1 ^a^	350.0 ± 204.3 ^a^	21.2 ± 17.9 ^a^
	Islands	13.4 ± 7.0 ^a^	119.4 ± 89.3 ^a^	21.2 ± 16.3 ^a^	223.9 ± 96.7 ^b,d^	23.5 ± 17.6 ^a^	66.1 ± 40.3 ^a^	26.3 ±15.0 ^a^	35.4 ± 31.2 ^a^	26.8 ± 7.9 ^b^	202.0 ± 260.4 ^a^	335.9 ± 206.8 ^a^	20.3 ± 19.3 ^a^
BF	No	12.3 ± 4.9 ^a^	108.7 ± 83.9 ^a^	24.7 ± 19.9 ^a^	196.2 ± 98.7 ^a^	24.5 ± 17.2 ^a^	73.3 ± 44.3 ^a^	45.4 ± 26.1 ^a^	48.4 ± 30.8 ^a^	22.12 ± 8.8 ^a^	133.4 ± 204.0 ^a^	414.8 ± 217.5 ^a^	24.3 ± 26.7 ^a^
	Yes	12.7 ± 6.3 ^a^	104.4 ± 99.8 ^a^	22.9 ± 16.8 ^a^	188.8 ± 132.2 ^a^	19.4 ± 15.3 ^b^	79.7 ± 45.2 ^a^	38.1 ± 23.3 ^b^	37.5 ± 23.7 ^b^	23.9 ± 11.8 ^a^	100.4 ± 112.3 ^a^	198.0 ± 151.0 ^b^	18.3 ± 16.3 ^a^
BMI	−5 to −1	12.7 ± 5.5 ^a^	102.8 ± 78.2 ^a^	26.1 ± 14.0 ^a^	182.0 ± 143.9 ^a^	28.6 ± 22.1 ^a^	84.3 ± 46.1 ^a^	42.9 ± 24.7 ^a^	46.5 ± 31.0 ^a^	21.8 ± 8.8 ^a^	70.0 ± 95.0 ^a^	283.6 ± 200.3 ^a^	24.26 ± 20.6 ^a^
	−1 to 1	12.3 ± 5.0 ^a^	107.4 ± 87.7 ^a^	22.3 ± 17.8 ^a^	197.8 ± 98.2 ^a^	21.6 ± 16.0 ^a^	73.2 ± 45.2 ^a^	42.6 ± 23.5 ^a^	44.2 ± 28 9 ^a^	23.2 ± 10.3 ^a^	142.3 ± 206.8 ^a^	359.6 ± 226.1 ^a,b^	23.3 ± 24.7 ^a^
	1 to 2	12.6 ± 6.1 ^a^	119.0 ± 100.9 ^a^	30.0 ± 26.7 ^a^	191.3 ± 108.4 ^a^	22.0 ± 12.6 ^a^	78.2 ± 44.7 ^a^	48.7 ± 33.3 ^a^	51.4 ± 31.2 ^a^	22.1 ± 9.1 ^a^	98.1 ±169.3 ^a^	426.0 ±208.2 ^b^	18.3 ± 12.6 ^a^
	> 2	13.6 ± 6.3 ^a^	91.6 ± 97.1 ^a^	23.5 ± 13.7 ^a^	204.0 ± 122.4 ^a^	26.3 ± 13.8 ^a^	64.5 ± 32.4 ^a^	36.0 ± 22.1 ^a^	36.5 ± 14.9 ^a^	21.1 ± 7.8 ^a^	238.0 ±270.4 ^a^	320.7 ± 247.2 ^a,b^	26.5 ± 28.9 ^a^

Data expressed as mean ± standard deviation. ^1^ Processed infant products were consumed such as infant formulas (mL/day), infant cereals (g/day), and infant puree (g/day). ^2^ Breast milk was not included in this category. ^3^ Milk and dairy products were consumed such as milk (mL/day), yogurt, dairy dessert, and cheeses (g/day). Different letters indicate significant differences between groups (*p* value ≤ 0.05). Independent samples *t*-test was used for intra-group comparison.

**Table 3:** Energy and macro- and micronutrient intake from diet compared with Dietary Reference Values (DRV) in the LAyDI cohort by age.

	DRV			18 Months (N 426)			DRV Compliance 18 Months (%)		24 Months (N 336)			DRV Compliance 24 Months (%)	
	AI	AR	RI	P25	Mean ± SD	P75	>AI	<AR	P25	Mean ± SD	P75	>AI	<AR
Energy (kcal/day)	-	-	-	899.4	1093.4 ± 291.7	1279.9	-	-	890.6	1094.2 ± 291.0	1281.8	-	-
Macronutrients							-	-				-	-
Fat (g/day) (kcal) (%)	-	-	35–40%	28.8	37.3 ± 11.5 (30.7)	44.6	-	-	30.2	38.3 ± 11.4 (31.5)	45.6	-	-
Saturated fatty acids (g/day)	ALAP	-	-	6.0	14.4 ± 9.2	21.3	-	-	8.3	15.5 ± 8.5	21.3	-	-
Monounsaturated fatty acids (g/day)	-	-	-	11.7	16.5 ± 6.1	19.6	-	-	13.3	17.7 ± 5.8 *	20.9	-	-
Polyunsaturated fatty acids (g/day)	-	-	-	3.7	4.8 ± 1.2	5.7	-	-	4.0	5 ± 1.5	6.2	-	-
ALA (g/day)	-	-	0.5	0.02	0.05 ± 0.05	0.06	-	-	0.02	0.05 ± 0.05	0.06	-	-
LA (g/day)	-	-	4	2.8	3.8 ± 1.2	4.5	-	-	3.1	3.9 ± 1.2	4.6	-	-
Carbohydrate (g/day) (kcal) (%)	-	-	45–60%	107.6	138.2 ± 43.9 (50.6)	163.14	-	-	105.3	136.2 ± 43.8 (49.8)	160.3	-	-
Dietary fiber (g/day)	10			11.7	15.9 ± 5.7	19.0	85		10.7	14.5 ± 5.5 *	17.4	81	
Protein (g/day) (kcal) (%)	-	-	-	40.0	50.3 ± 15.9 (18.4)	60.2	-	-	39.9	50.4 ± 15.9 (18.4)	60.4	-	-
Animal protein (g/day)	-	-	-	21.6	31.4 ± 13.5	38.9	-	-	23.3	32.2 ± 12.6	40.6	-	-
Vegetal protein (g/day)	-	-	-	11.7	16.2 ± 6.6	19.5	-	-	11.4	16.4 ± 6.7	20.0	-	-
Micronutrients													
Vitamin A (μg RAE/day)	-	205	-	431.6	796.7 ± 478.5	990.6	-	5.4	369.9	684.7 ± 466.9 *	834.9	-	6.2
Thiamin (mg/day)	-	0.072	-	0.8	1.0 ± 0.3	1.2	-	0	0.7	0.9 ± 0.3 *	1.1	-	0
Riboflavin (mg/day)	-	0.5	-	1.0	1.4 ± 0.6	1.7	-	3.8	0.9	1.3 ± 0.5	1.7	-	3.6
Niacin (mg/day)	-	1.3	-	8.8	12.3 ± 4.7	14.8	-	0.2	8.6	11.5 ± 4.2 *	13.9	-	0
Vitamin B-6 (mg/day)	-	0.5	-	1.2	1.6 ± 0.5	1.9	-	1.2	1.1	1.4 ± 0.5 *	1.7	-	0.3
Folate (μg DFE/day)	-	90	-	357.8	555.3 ± 279.5	698.37	-	0.2	343.4	496.9 ± 222.0 *	632.5	-	0.3
Vitamin B-12 (μg/day)	1.5		-	1.9	2.74 ± 1.3	3.4	85.2	-	1.9	2.8 ± 1.3	3.7	84.2	-
Vitamin C (mg/day)		15	-	78.6	125.5 ± 63.9	159.2	-	0	64.8	109.3 ± 59.7 *	137.8	-	0.3
Vitamin D (μg/day)	15	-	-	0.7	2.9 ± 2.9	3.7	0.7	-	0.7	2.1 ± 2.3 *	2.7	0	-
Vitamin E (mg/day)	6	-	-	4.2	6.2 ± 4.0	7.0	38.7	-	3.9	5.4 ± 3.0 *	6.0	25.6	-
Calcium (mg/day)	-	390	-	440.9	665.3 ± 294.5	862.2	-	20.2	418.7	643.5 ± 281.4	846.6	-	22
Phosphorus (mg/day)	250	-	-	672.5	889.5 ± 306.4	1088.5	99.1	-	661.4	871.7 ± 285.8	1071.8	99.4	-
Iron (mg/day)	-	5	-	6.0	8.4 ± 3.2	10.3		12.2	5.6	7.6 ± 2.7 *	9.0	-	15.8
Magnesium (mg/day)	170	-	-	162.8	210.4 ± 68.5	256.3	70.9	-	160.9	199.9 ± 60.7 *	235.7	67.9	-
Manganese (mg/day)	0.5	-	-	1.6	2.4 ± 1.0	2.9	99.8	-	1.6	2.2 ± 0.9 *	2.7	99.7	-
Zinc (mg/day)	-	3.6	-	4.7	7.0 ± 3.0	9.1	-	11.7	5.1	6.9 ± 2.6	8.6	-	8.9

Values are presented as percentiles, mean ± standard deviation, or percentage of the reference intake range (RI) compliance based on intakes from the European Food Safety Authority (EFSA). AI, adequate intake. ALA, alpha-linolenic acid. ALAP, as low as possible. AR, average requirement. LA, linoleic acid. RI, reference intake range. * *p* value for difference between 18 and 24 months from *t*-test.

**Table 4:** Differences in bioactive compound intakes according to age in the LAyDI cohort.

	18 Months			24 Months		
Bioactive Compounds	Mean ± SD	P25	P75	Mean ± SD	P25	P75
Total phenols (mg/day)	659.4 ± 335.0	436.4	830.0	654.2 ± 322.5	432.0	842.8
Total (poly)phenols (mg/day) ^1^	153.7 ± 84.1	97.3	195.4	151.0 ± 81.7	97.6	188.2
Flavonoids (mg/day)	109.2 ± 67.2	62.3	142.3	104.9 ± 61.4	63.4	134.3
Anthocyanins	23.9 ± 23.1	8.6	29.5	25.3 ± 28.6	6.7	3.7
Dihydrochalcones	1.7 ± 1.0	1.1	1.7	1.8 ± 1.0	1.1	2.2
Flavanols	42.2 ± 31.9	17.3	55.9	42.8 ± 31.0	20.7	56.5
Flavanones	27.9 ± 25.3	6.8	38.3	27.8 ± 25.4	4.0	41.7
Flavones	0.6 ± 0.8	0.2	0.7	0.5 ± 0.8	0.1	0.5
Flavonols	19.0 ± 26.8	7.1	20.3	14.0 ± 11.0 *	7.2	17.8
Isoflavanoids	0.7 ± 2	0.08	0.6	0.8 ± 2.9	0.1	0.6
Phenolic acids (mg/day)	40.9 ± 21.9	26.2	53.5	42.0 ± 23.0	25.8	53.7
Lignans (mg/day)	0.1 ± 0.2	0.02	0.3	0.1 ± 0.2	0.02	0.3
Non-phenolic metabolites (mg/day)	1.7 ± 0.7	1.2	2.1	1.8 ± 0.7	1.3	2.3
Other polyphenols (mg/day)	4.3 ± 2.5	3.3	4.9	4.8 ± 2.8	3.3	6.8
Dietary fiber (g/day)	15.7 ± 5.7	11.7	19.0	14.5 ± 5.5 *	10.7	17.4
Insoluble	10.4 ± 4.3	7.4	12.7	9.3 ± 4.03 *	6.4	11.5
Soluble	2.1 ± 1.0	1.5	2.5	1.9 ± 0.9 *	1.3	2.2

Values are presented as mean ± standard deviation and percentiles. ^1^ Total (poly)phenols were calculated as the sum of total flavonoids, phenolic acid, lignans, non-phenolic metabolites, and other polyphenols. * *p* value for difference between 18 and 24 months from *t*-test.

## Data Availability

Not applicable.

## Supplementary Materials

The following are available online at https://www.mdpi.com/1660-4601/18/3/939/s1, Figure S1: Geographical distribution of the sample, Table S1: Food group description.
