# Supplementary material for: Identification of Nutritional Targets in Spanish Children Belonging to the LAyDI Cohort for the Development of Health Promotion Strategies in the First Two Years of Life
Source: Int J Environ Res Public Health. 2021 Jan 22;18(3):939. doi: 10.3390/ijerph18030939 (PMC7908140; doi:10.3390/ijerph18030939)
Supplement: Supplementary file 1 [file ijerph-18-00939-s001.zip › ijerph-1054315-supplementary/G¿«mez et al-Supplementary material.docx]

**Supplementary Table S1.** Food group description

| **Food group** | **Components** |
| --- | --- |
| Oils | Vegetable oils and solid fats |
| Vegetables | Bulbs, mushrooms, roots, inflorescences and stem and leaf vegetables |
| Legumes | Lentils, chickpeas, beans and peas |
| Fruit | Fresh, dried, and canned fruits |
| Potato and tubers | Potato and sweet potato |
| Cereals and cereal products | Bread, pasta, flours and grains |
| Meat and meat products | Poultry, red meat, processed meat and others |
| Fish | Fish and fish products, crustaceans and mollusks |
| Eggs | Eggs |
| Processed infant products | Infant formulas, infant cereals and infant puree |
| Milk and dairy products | Milk, yogurt, dairy dessert and cheeses |
| Sweets and desserts | Sweets, cakes, biscuits, chocolate and honey |

EPIC classification [29] and Gómez-Martín et al. [35].
