# Supplementary figures and images for: Identification of Nutritional Targets in Spanish Children Belonging to the LAyDI Cohort for the Development of Health Promotion Strategies in the First Two Years of Life
Source: Int J Environ Res Public Health. 2021 Jan 22;18(3):939. doi: 10.3390/ijerph18030939 (PMC7908140; doi:10.3390/ijerph18030939)

## Slide 1
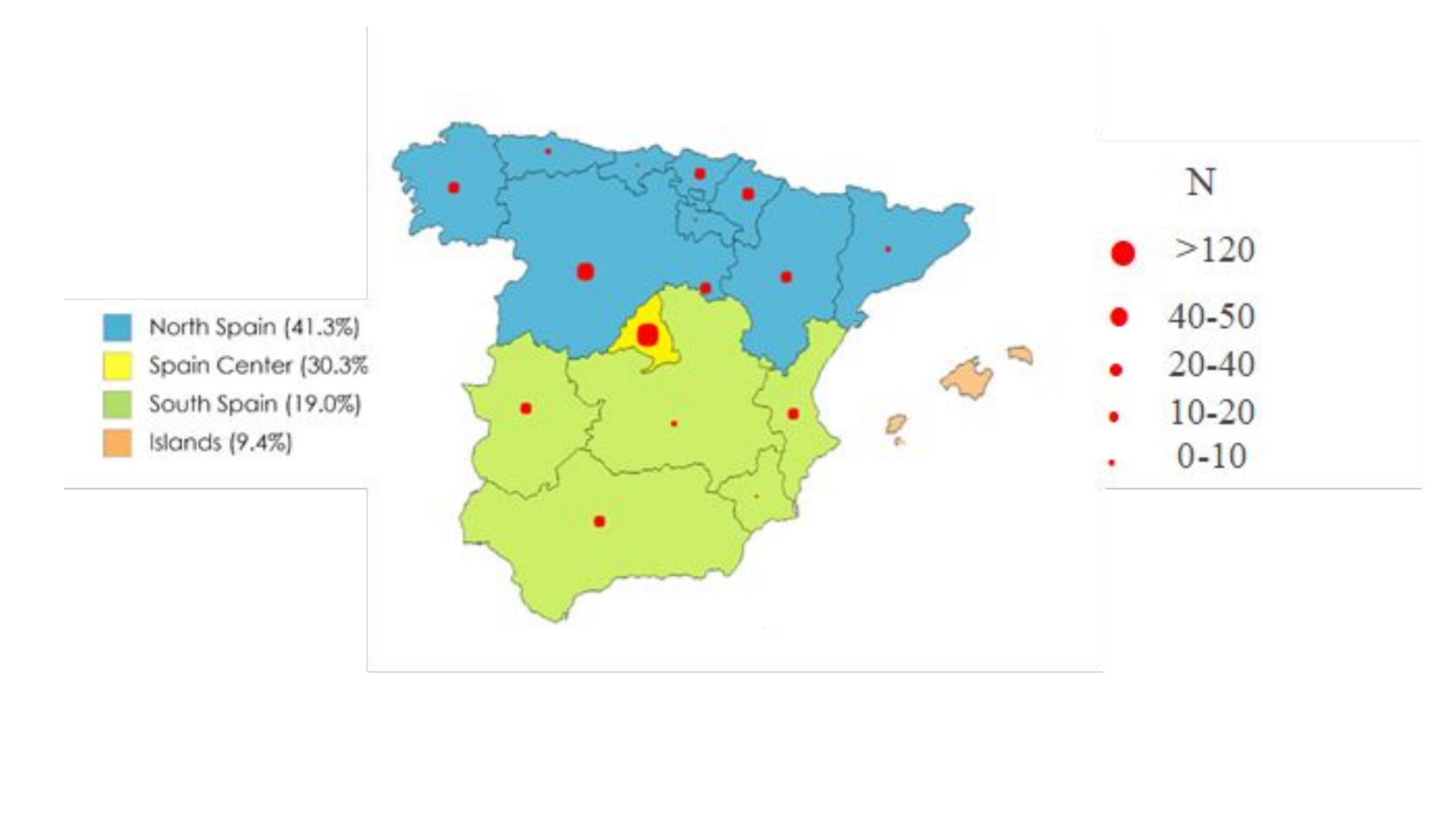

Supplement: Supplementary file 1 [file ijerph-18-00939-s001.zip › ijerph-1054315-supplementary/G¿«mez-Mart¿¬n et al Supplementary Figures.pptx]
